# Does the midsole matter in the tribological performance of footwear?

**DOI:** 10.1080/19424280.2025.2489736

**Published:** 2025-06-20

**Authors:** Kitti Csajbók, Alex Klein-Paste, Viveca Wallqvist

**Affiliations:** a Department of Civil and Environmental Engineering, Norwegian University of Science and Technology (NTNU), Trondheim, Norway; b Department of Sustainable Materials and Packaging, Research Institutes of Sweden (RISE), Stockholm, Sweden

**Keywords:** Friction, ice, footwear, TPU, midsole

## Introduction

Tribology, the study of surfaces in relative motion, is key to footwear slip resistance. Research often targets outsole materials, but overall performance is more complex and depends on the shoe’s dynamic, mechanical, and thermal properties. The shoe’s vertical composition, including the midsole and outsole, greatly affects performance, especially on ice, as the temperature dependence of the dynamic performance of materials becomes critical (Beschorner et al., 2022; Persson, 2001).

## Purpose of the study

The purpose of this study is to explore how the dynamic tribological performance of footwear materials is influenced by changes in the vertical composition, particularly changes in the hardness of the midsole material while using the same outsole coating.

## Methods

**Figure 1.**
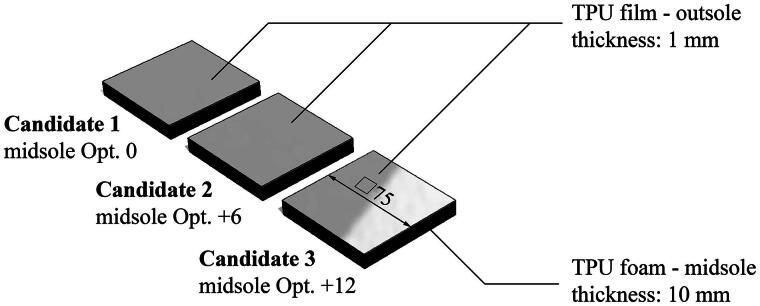
Samples (candidate 1–3) with the same unpatterned TPU film as a coating but with three different TPU foams as a base (with opt. 0, +6, +12 hardness respectively).

Three samples were prepared (Figure 1) with different hardness of the midsole material (0, 6 and 12 opt) while the same thermoplastic polyurethane (TPU) film was used as the outer coating. As the interacted surface was the same TPU film, the mechanical, chemical and topological properties of the surfaces were identical. Meanwhile, the overall performance differs due to the different hardness of the TPU foams. The relative grip resistance of the samples was tested with a portable walkway tribometer (Mark IIIB). The test procedure was the following: an asphalt sample was frozen at −10°C, and a layer of ice was applied to the top with a brush in a 0°C laboratory. The sample was tested ten times, followed by ten tests with a reference, then ten more with the original sample. The relative grip was calculated as the ratio of the average candidate/average reference grip. The procedure was repeated for all three samples on new asphalts. A reference was needed to ensure consistent surface conditions, using a standardized Neolite Slider test foot (intended use with (ASTM International, 2016)), which is a styrene-butadiene rubber (SBR) with a hardness of Shore 92 A.

## Results

A winter shoe material has a better grip if the candidate/reference grip (relative grip) is the highest. Results (Table 1) show that the relative grip resistance was highest for the material with the softest midsole and the lowest overall hardness. The lowest grip was found in the sample with the hardest midsole and highest overall hardness.

**Table 1.** Relative grip and shore a hardness of the samples.

Candidate	ShoreA hardness at 0 °C	Relative grip resistance
1	50.3 ± 0.97	2.10
2	52.6 ± 0.89	1.79
3	54.4 ± 0.96	1.31

## Discussion and conclusion

The generalization that softer shoe materials always provide higher grip is not entirely accurate due to the design and material complexity. In a detailed tribological test, multiple parameters interact, creating a combined effect that complicates straightforward conclusions. However, by isolating these parameters, we can measure the individual impacts, which in our case shows that the harder the sample, the lower the relative grip is. Our results reveal that the midsole material can be a significant factor in the overall tribological performance of the shoes. Choosing the right midsole can nearly double the grip on ice, highlighting the impact of vertical components on footwear tribology.
